# Cyclin K interacts with β-catenin to induce Cyclin D1 expression and facilitates tumorigenesis and radioresistance in lung cancer

**DOI:** 10.7150/thno.42578

**Published:** 2020-09-11

**Authors:** Guojun Yao, Jing Tang, Xijie Yang, Ye Zhao, Rui Zhou, Rui Meng, Sheng Zhang, Xiaorong Dong, Tao Zhang, Kunyu Yang, Gang Wu, Shuangbing Xu

**Affiliations:** Cancer Center, Union Hospital, Tongji Medical College, Huazhong University of Science and Technology, Wuhan 430022, China.

**Keywords:** Cyclin K, β-catenin, Cyclin D1, radioresistance, lung cancer

## Abstract

**Rationale:** Radioresistance remains the major cause of local relapse and distant metastasis in lung cancer. However, the underlying molecular mechanisms remain poorly defined. This study aimed to investigate the role and regulatory mechanism of Cyclin K in lung cancer radioresistance.

**Methods:** Expression levels of Cyclin K were measured by immunohistochemistry in human lung cancer tissues and adjacent normal lung tissues. Cell growth and proliferation, neutral comet and foci formation assays, G2/M checkpoint and a xenograft mouse model were used for functional analyses. Gene expression was examined by RNA sequencing and quantitative real-time PCR. Protein-protein interaction was assessed by immunoprecipitation and GST pull-down assays.

**Results:** We report that Cyclin K is frequently overexpressed and correlates with poor prognosis in lung cancer patients. Functionally, we demonstrate that Cyclin K depletion results in reduced proliferation, defective G2/M checkpoint and enhanced radiosensitivity in lung cancer. Mechanistically, we reveal that Cyclin K interacts with and promotes the stabilization of β-catenin protein, thereby upregulating the expression of Cyclin D1. More importantly, we show that Cyclin D1 is the major effector that mediates the biological functions of Cyclin K in lung cancer.

**Conclusions:** These findings suggest that Cyclin K positively modulates the β-catenin/Cyclin D1 axis to promote tumorigenesis and radioresistance in lung cancer, indicating that Cyclin K may represent a novel attractive biomarker for lung cancer radiotherapy.

## Introduction

Lung cancer remains the predominant cause of cancer morbidity and death worldwide 1. Non-small cell lung cancer (NSCLC), which consists of adenocarcinoma, squamous cell carcinoma and large cell carcinoma, comprises approximately 85% of total cases 2. Currently, radiotherapy is required for treatment benefit in more than half of lung cancer patients. Despite advances in radiotherapy devices and techniques, the therapeutic effects remain less than satisfactory. The intrinsic radioresistance of lung cancer cells limits the clinical efficacy of radiotherapy, leading to tumor recurrence and distant metastasis 3. Accordingly, it is extremely important to identify novel biomarkers and to elucidate the molecular mechanisms of radioresistance in lung cancer.

The cell cycle controls the life span of normal cells, and dysregulation of the cell cycle may result in unlimited cell proliferation, which has the potential for cancerization 4, 5. Control of the cell cycle is generally maintained by three different kinds of proteins, termed CDKs (Cyclin-dependent kinases), cyclins and CKIs (CDK inhibitors) 6. Normally, cyclins bind to CDKs and regulate kinase activity in a timely manner. Among them, cyclin K, which is most similar to cyclins C and H, is considered a new member of the transcriptional cyclin family 7. Initially, Cyclin K was proposed to play dual roles in controlling CDK and RNA polymerase II (RNAP II) activity 7. It is becoming increasingly apparent that Cyclin K forms functional complexes with CDK9, CDK12 and CDK13, implicating this protein in the regulation of gene transcription and DNA damage response, as well as in the maintenance of embryonic stem cell pluripotency and genomic stability 8-12. For instance, Cyclin K has been reported to associate with CDK9 to activate transcription via RNA 8. In addition, it was reported that the Cyclin K/Cdk12 complex maintains genomic integrity and represents a master regulator of DNA damage response genes in various types of cancer 12-14. These results strongly suggest that Cyclin K is closely correlated with tumor cell radiosensitivity. However, the role and regulatory mechanism of Cyclin K in lung cancer radioresistance have not been characterized.

Wnt/β‑catenin signaling is well known to be involved in the control of cell cycle progression, cell proliferation and differentiation, migration and invasion, genetic stability and stem cell survival, and is associated with various diseases, including cancer, metabolic disease and neurodegenerative disorders 15, 16. In this signaling pathway, β‑catenin functions as a transcriptional co-activator of Wnt target genes, such as c-Myc, Axin-2 and cyclin D1 17. Interestingly, a growing body of evidence demonstrates that deregulation of Wnt/β‑catenin signaling is associated with tumor radioresistance in several types of human cancers, including breast cancer, head and neck cancer, prostate cancer, glioblastoma and colorectal cancer 18-22. For example, ALDH1A1 is induced by Wnt signaling and results in radioresistance in prostate cancer progenitor cells 21. Furthermore, it has been proposed that Wnt/β‑catenin signaling transactivates and enhances expression of LIG4, a key enzyme for non-homologous end joining (NHEJ), leading to radioresistance in colorectal cancer cells 22. These data suggest that activation of Wnt/β‑catenin signaling may facilitate DNA damage repair capacity, highlighting the essential role of Wnt/β‑catenin signaling in cancer radioresistance.

In our research, we show that Cyclin K is overexpressed and promotes tumorigenesis and radioresistance in lung cancer both *in vitro* and *in vivo*. We further reveal that Cyclin K associates with β‑catenin and induces the expression of Cyclin D1, which is a critical downstream Wnt target gene. More importantly, Cyclin K exerts its biological functions in a β‑catenin/Cyclin D1-dependent manner.

## Materials and Methods

### Cell culture

Human lung cancer cell lines A549 and H460, as well as HEK293T cells, were purchased from American Type Culture Collection and maintained in Dulbecco's modified Eagle medium (DMEM) containing 10% fetal bovine serum. All cell lines were tested for mycoplasma contamination and authenticated using short tandem repeat (STR) profiling.

### Constructs

Flag-Cyclin K, Flag-Cyclin D1 and Flag-β-catenin plasmids were purchased from Vigene biosciences. The Flag-Cyclin K plasmid was subcloned into the pDONR201 entry vector and transferred to the destination vector using the indicated SFB tag with Gateway Technology (Invitrogen). The Cyclin K siRNA resistant plasmid (SFB-Cyclin K-R) was generated by introducing seven nucleotide substitutions and was verified by DNA sequencing.

### RNA interference

A549 and H460 cells were transfected with 50 nM siRNAs using Lipofectamine RNAiMAX reagent (Invitrogen) for 48 h. The sequences of oligonucleotides targeting Cyclin K are as follows: siCyclin K#1, 5′-AAGCAACUCAAAGGUGAUAAA-3′, which has been described previously 9; siCyclin K#2, 5′-CAAGUUUGAUUUACAGGUA-3′; siCyclin D1, 5′-ACACCAAUCUCCUCAACGAUU-3′; Scramble siRNA, 5′-UUCUCCGAACGUGUCACGU-3′.

### Establishment of Cyclin K stable knockdown cells

This assay was performed as previously described 23, 24. Briefly, HEK293T cells were transfected with a combination of Cyclin K shRNA, pSPAX2 and pMD2G plasmids. The supernatants were filtered 48 h after transfection and equally mixed with RPMI-1640 media to infect H460 cells. Next, 10 μg/mL polybrene was added to improve infection efficiency. Stable cell lines were selected with media containing 2 μg/ml puromycin and confirmed by Western blotting. shRNA sequences are as follows: shCyclin K#1, 5′-AAGCAACTCAAAGGTGATAAA-3′; shCyclin K #2, 5′-CAAAGCAACTCAAAGGTGA-3′; Control shRNA, 5′-TTCTCCGAACGTGTCACGT-3′.

### Western blotting and immunoprecipitation

A549 and H460 cells were lysed in NETN (20 mM Tris-HCl [pH 8.0], 1 mM EDTA, 100 mM NaCl, and 0.5% Nonidet P-40) for 30 min at 4℃ followed by centrifugation (14,000 rpm for 20 min). Supernatants were denatured in 5 × SDS buffer at 95℃ for 5 min followed by SDS-PAGE, then transferred to polyvinylidene difluoride membranes (Bio-Rad). Membranes were subsequently incubated with the relevant primary antibody, followed by an appropriate secondary antibody. Protein expression was detected by immunoblotting using the following antibodies: anti-Cyclin K (A301-939A, Bethyl Laboratories), anti-β-catenin (#8480, Cell Signaling Technology), anti-Cyclin D1 (#55506, Cell Signaling Technology), anti-GAPDH (sc-365062, Santa Cruz Biotechnology), anti-Flag (F1804, Sigma-Aldrich) and anti-GST (#2624, Cell Signaling Technology).

For exogenous immunoprecipitation, cells were transfected with the SFB-Cyclin K plasmid using Lipofectamine 2000 reagent (Invitrogen) for 24 h, then lysed and incubated with S beads (69704, Millipore) overnight. For endogenous immunoprecipitation, cell lysates were first incubated with anti-Cyclin K antibody followed by protein A/G agarose (sc-2003, Santa Cruz Biotechnology) overnight. Precipitates were washed four times with NETN buffer and analyzed by Western blotting.

### GST pull-down assay

The GST-Cyclin K fusion protein was immobilized on GST beads (GE Healthcare) and incubated with lysates prepared from HEK293T cells that were transiently transfected with the Flag-β-catenin plasmid overnight at 4°C. Samples were centrifuged and washed four times and then detected by Western blotting.

### RNA transcriptome sequencing (RNA-seq) assay

Total RNA from experimental cells was extracted using TRIzol Reagent (Invitrogen). All samples were sequenced by Beijing CapitalBio Technology Inc. Briefly; sequencing was performed using an Illumina Hiseq Xten System PE150. Differentially expressed genes were identified using the StringTie (1.3.3b) software. Gene and transcript FPKM (Fragments per Kilobase Million) were then calculated, and genes were selected that exhibited a fold change > 3 and p value < 0.01. The RNA-seq data have been deposited in the Gene Expression Omnibus database under accession number GSE143147 (https://www.ncbi.nlm.nih.gov/geo).

### Quantitative real-time PCR

Total RNA was extracted using TRIzol Reagent (Invitrogen) based on the manufacturer's instructions. First-strand cDNA was synthesized using qPCR RT Master Mix (Toyobo). Expression of specific mRNAs was determined using SYBR Green real-time PCR kit (Toyobo) with GADPH as a loading control. The ΔCt method was used to measure relative gene expression levels. Primer sequences are listed in Table S1.

### Cell growth and proliferation assays

For the cell growth assay, after transfection, cells were plated in 6-well plates at 1 × 10^4^ cells per well. After 1, 3, 5, and 7 days, cells were collected and counted under a microscope. For cell proliferation analysis, fourteen days after seeding, cells were fixed in 10% formalin and stained with crystal violet for 30 min, and the surviving colonies (a colony containing more than 50 cells) were counted.

### CFSE proliferation assay

A549 and H460 cells were incubated in RPMI-1640 containing 10 μM/mL CFSE solution on ice for 10 min, and further steps were performed following the manufacturer's procedures (CellTrace™ CFSE Cell Proliferation Kit, Thermo Fisher).

### G2/M checkpoint assay

This assay was performed as previously described 25. A549 and H460 cells were first treated with 6 Gy irradiation followed by incubation with 100 ng/mL nocodazole for 20 h and subsequent collection. After fixation, cells were permeabilized in 0.5% Triton X-100 in PBS and then incubated with phospho-histone H3 (#3465, Cell Signaling Technology) for 2 h, followed by incubation with secondary antibody. Cells were stained with propidium iodide, and cellular fluorescence was determined by flow cytometry.

### Clonogenic cell survival assay

A549 and H460 cells were transfected with indicated siRNAs for 48 h and then plated in triplicate in six-well plates at various cell densities. Cells were exposed to irradiation with doses ranging from 2 to 8 Gy using an X-ray irradiator (Varian, USA) at a dose rate of 6 Gy/min. After fourteen days, surviving colonies (a colony containing more than 50 cells) were counted under the microscope.

### Neutral comet assay

The neutral comet assay was performed according to the manufacturer's instructions. Briefly, A549 and H460 cells transfected with the indicated siRNAs for 48 h were exposed to irradiation at 6 Gy. Four hours later, cells were digested in trypsin, washed, and then plated to CometSlide^TM^ (Trevigen) with a comet LMAgarose mix. Cells were stained by SYBR Gold Staining Solution and imaged using a fluorescence microscope.

### Immunofluorescence staining

A549 and H460 cells transfected with the indicated siRNAs were cultured on coverslips and then exposed to irradiation. Four hours after irradiation, cells were fixed in 4% paraformaldehyde, permeabilized, and incubated with anti-γ-H2AX (ab26350, Abcam) or anti-Rad51 (ab133534, Abcam) overnight, followed by Alexa Fluor-conjugated fluorescent secondary antibodies and DAPI staining. Images were visualized and captured by confocal microscopy.

### *In vivo* xenograft mouse model

The Medical Ethics Committee of Tongji Medical College, Huazhong University of Science and Technology approved all animal experiments. Nude mice (BALB/c, 4-5 weeks old) were purchased from Beijing Vital River Laboratory Animal Technology (Beijing, China). For tumorigenesis, nude mice were randomly divided into three groups (Shcontrol, ShCyclin K#1 and ShCyclin K#2) and then subcutaneously injected in the right posterior limb with 5 × 10^6^ H460 cells (five mice per group). For the radioresistance assay, mice were randomly classified into four groups (Shcontrol, Shcontrol+IR, ShCyclin K#1+IR, and ShCyclin K#2+IR), and tumor xenografts were subjected to 10 Gy local irradiation using an X-ray irradiator (Varian, USA) at a dose rate of 6 Gy/min when tumors reached a calculated average volume of 100 mm^3^, which was previously described 26. Tumor sizes were measured every three days using calipers. Tumor volumes were calculated using the following formula: V= (Length × Width^2^)/2 and tumor weights were measured and recorded.

### Tissue microarray and immunohistochemical (IHC) staining

A lung cancer tissue microarray was obtained from Shanghai Outdo Biotech (Shanghai, China) containing 89 lung cancer tissues and paired para-carcinoma tissues. IHC analysis was performed as previously described 26-28. Briefly, tissue samples were fixed in 10% formalin and processed for paraffin embedding. After deparaffinization, rehydration, antigen retrieval, and blocking endogenous peroxidase activity, sections were incubated with anti-Cyclin K (A301-939A, Bethyl Laboratories) and anti-Ki67 (ab15580, Abcam) antibodies overnight at 4°C. Sections were incubated with secondary antibodies for 1 h and counterstained with hematoxylin for 30 s before dehydration and mounting. Images were taken by a microscope scanner. Both staining intensity and positive percentage were utilized to detect the expression of Cyclin K in lung cancer tissue.

### Statistical analyses

All data are presented as the mean values ± SD unless otherwise specified from at least three independent experiments. Differences between two separate groups were evaluated by two-tailed Student's *t*-test. χ^2^ test was utilized to evaluate the correlation between Cyclin K expression and clinicopathological variables. Overall survival curves were generated using the Kaplan-Meier method. *P*-values < 0.05 were considered statistically significant.

## Results

### Cyclin K is frequently overexpressed and predicts poor prognosis in lung cancer

**Figure 1:**
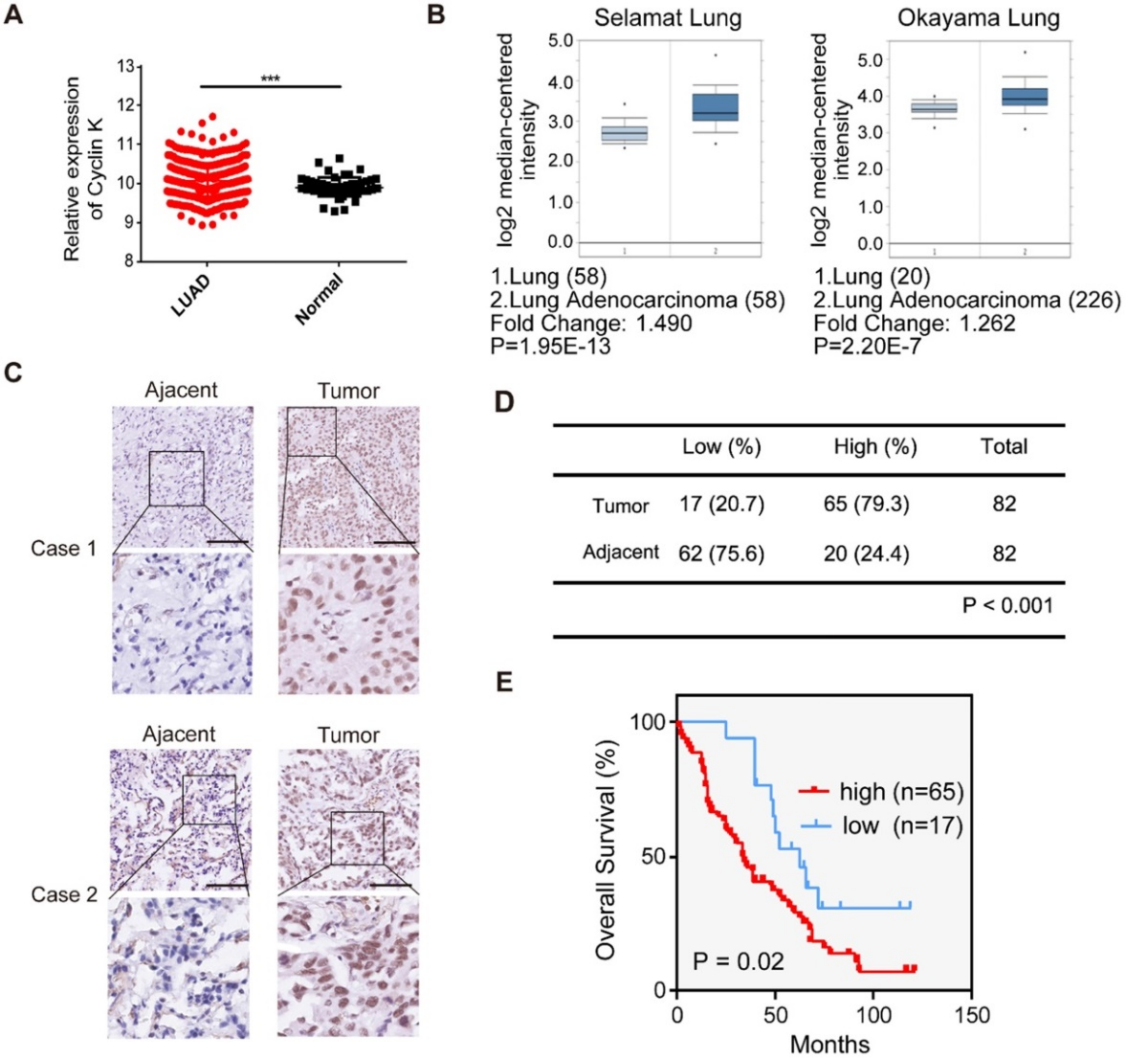
**Cyclin K is overexpressed and predicts poor prognosis in lung cancer patients. (A)** Comparison of the Cyclin K mRNA levels between tumor and normal tissues obtained from TCGA lung adenocarcinoma (LUAD) dataset. ***P < 0.001. **(B)** Box plots showing Cyclin K mRNA levels are upregulated in LUAD tissues compared to normal lung tissues obtained from the Oncomine database.** (C)** Representative immunohistochemical staining images for Cyclin K in lung cancer and adjacent lung tissues. Scale bar, 50 µm.** (D)** Statistical analysis of immunohistochemical staining for Cyclin K in lung adenocarcinoma tissue microarray. **(E)** OS (overall survival) curve indicating that high expression of Cyclin K predicts poor prognosis in lung cancer patients.

To investigate Cyclin K expression in lung cancer, we first analyzed Cyclin K mRNA levels in lung adenocarcinoma and adjacent normal lung tissue using TCGA and the Oncomine database. Cyclin K expression was upregulated in lung cancer tissues compared to normal lung tissues (Figure 1A-B). To further examine whether Cyclin K expression was indeed increased, we measured Cyclin K protein levels in lung adenocarcinoma tissue arrays. As shown in Figure 1C-D, Cyclin K was overexpressed in lung cancer tissues compared to adjacent normal tissues. Moreover, higher expression of Cyclin K was significantly associated with poor overall survival (*p* < 0.05) (Figure 1E). There was also a significant correlation between Cyclin K expression and tumor stage in clinicopathological parameters (*p* < 0.05) (Table S2). These results suggest that Cyclin K may act as a tumor promoter and that its upregulation may contribute to the development and progression of lung cancer.

### Cyclin K silencing suppresses tumorigenesis in lung cancer both *in vitro* and *in vivo*

**Figure 2:**
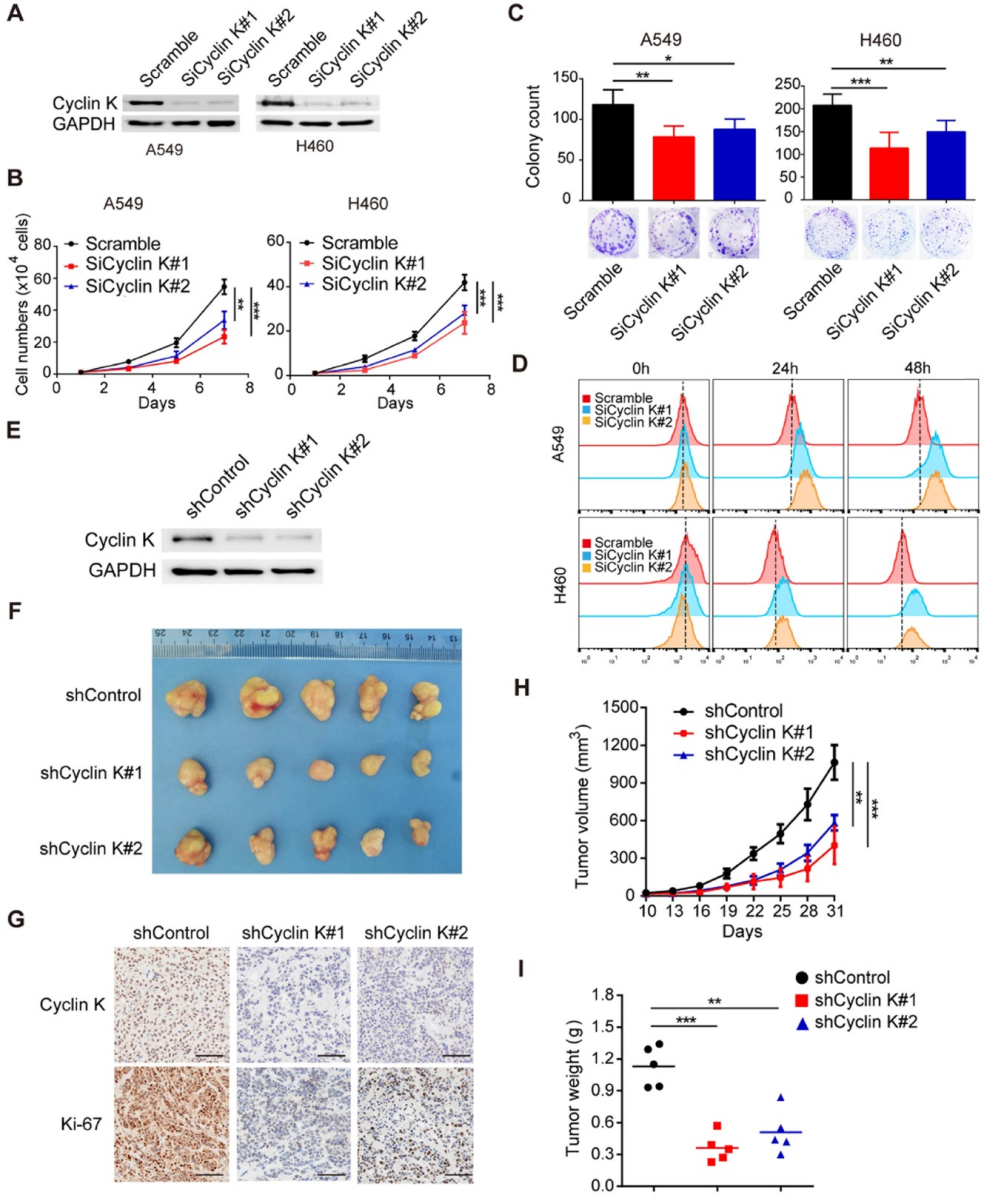
**Cyclin K knockdown suppresses tumorigenesis in lung cancer both* in vitro* and *in vivo*. (A)** Cyclin K was successfully knocked down by two different siRNAs. A549 (left) or H460 (right) cells were transfected with the indicated siRNAs for 48 h, then collected and analyzed by Western blotting (n = 3). **(B)** Cyclin K depletion decreased cell growth. A549 and H460 cells were transfected with indicated siRNAs for 48 h and then seeded. Cell numbers were calculated every two days (n = 3). **(C)** Colony formation was significantly decreased in Cyclin K-depleted cells. A549 and H460 cells transfected with indicated siRNAs were grown for two weeks. Colonies were counted and are shown as histograms (n= 3). *P < 0.05, **P < 0.01, ***P < 0.001 compared to control cells. **(D)** Cells were transfected with indicated siRNAs for 48 h, then dyed with CFSE stain. Cells were harvested in trypsin at indicated time points and analyzed by flow cytometry (n = 3).** (E)** H460 cells stably transfected with shCyclin K or shControl were lysed and analyzed by Western blotting (n = 3). **(F)** Cyclin K knockdown alleviated tumor growth *in vivo*. Representative images of xenograft tumors are shown (five mice/group). **(G)** Representative immunohistochemistry images showing Cyclin K and Ki-67 expression in xenograft tumors in the three groups. Scale bar, 50 µm. **(H)** Growth curves of tumors in the three groups are presented (five mice/group). Data are shown as the mean tumor volume ± SEM.** (I)** Tumor weight in the three groups are shown (five mice/group).

Due to the overexpression of Cyclin K in lung cancer, we speculated that Cyclin K might function as an oncoprotein. To test this hypothesis, we first knocked down the expression of Cyclin K in two different lung cancer cell lines using siRNAs (Figure 2A) and found that depletion of Cyclin K impaired tumor cell growth and colony formation ability (Figure 2B-C). In keeping with this notion, the CFSE proliferation assay provided additional compelling evidence that Cyclin K silencing inhibited cell proliferation (Figure 2D). To further investigate whether Cyclin K knockdown suppresses tumorigenesis *in vivo*, a nude mouse xenograft experiment was performed. As shown in Figure 2E-I, both tumor size and weight from mice injected with Cyclin K stable knockdown lung cancer cells were substantially decreased compared to those injected with control cells. Taken together, these data support the oncogenic role of Cyclin K and indicate that Cyclin K silencing suppresses tumorigenesis in lung cancer both *in vitro* and *in vivo*.

### Cyclin K participates in G2/M checkpoint control and DNA damage response

**Figure 3:**
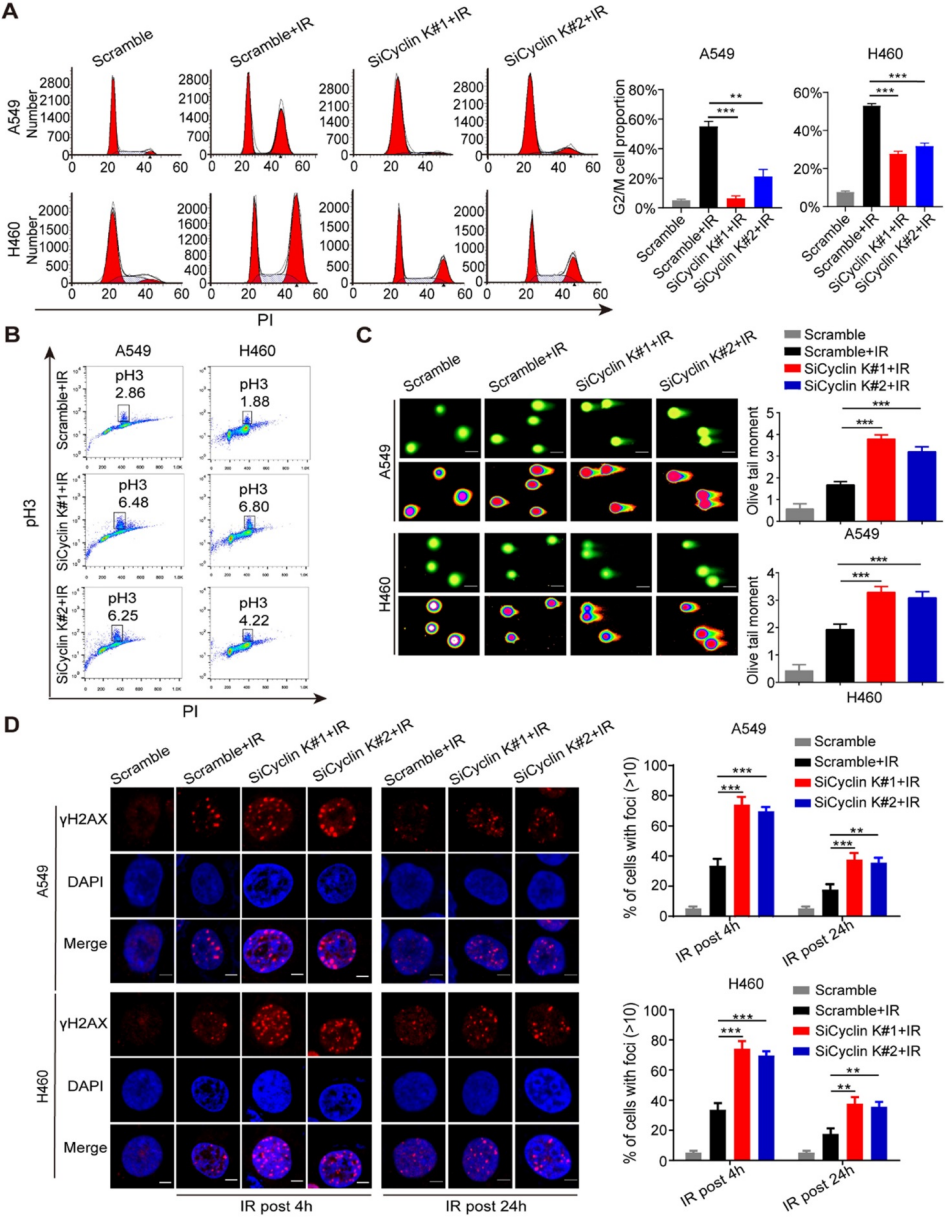
** Cyclin K participates in G2/M checkpoint control and the DNA damage response. (A)** Cyclin K silencing abrogated IR-induced G2/M arrest. Cells were transfected with indicated siRNAs for 24 h and then exposed to IR. After another 24 h, cells were harvested and analyzed by flow cytometry. **P < 0.01. ***P < 0.001 (n = 3). **(B)** Cyclin K depletion abolished the G2/M checkpoint. Cells transfected with siRNA and then exposed to IR were treated with nocodazole (100 ng/mL) for 22 h before being harvested. The samples were analyzed by flow cytometry. The percentage of cells positive for phospho-histone H3 (pH3) is indicated (n = 3). **(C)** Cells were transfected with siRNA and then exposed to IR. After 4 h, cells were harvested and seeded to comet slides, then lysed for electrophoresis. ***P < 0.001 (n = 3). Scale bar, 10 µm. **(D)** Left panel: Cells transfected with indicated siRNAs were irradiated and then harvested after 4 h or 24 h. Representative immunostaining images are shown. Right panel: Quantification results of γ-H2AX foci are also shown. Scale bar, 10 µm. **P < 0.01. ***P < 0.001 (n = 3).

Previous studies have indicated that the Cyclin K/Cdk12 complex may be involved in the regulation of the DNA damage response (DDR) 10-12. However, there is no direct evidence showing the role of Cyclin K in this process. As shown in Figure S1, depletion of Cyclin K has no significant effect on cell cycle distribution under non-irradiation conditions. However, IR-induced G2/M arrest was significantly abolished in Cyclin K depleted cells (Figure 3A). To discriminate between cells in G2 and M phase, phospho-Ser10-histone H3 (pH3), an identified biomarker for M phase, was measured by flow cytometry. As shown in Figure 3B, the percentage of cells marked with pH3 was increased in response to Cyclin K knockdown. These results suggest that Cyclin K depletion abolishes the G2/M checkpoint after DNA damage. In agreement with the above results, the comet assay clearly showed that cells transfected with Cyclin K siRNAs had greater olive tail moment than control cells upon DNA damage (Figure 3C), indicating that more DNA damage occurred in Cyclin K deficient cells. It is well established that the formation of γ-H2AX foci represents another readout of the DNA damage response. In line with the results from the G2/M checkpoint and comet assay, DNA damage-induced γ-H2AX foci formation was increased when Cyclin K was knocked down (Figure 3D). Therefore, these data provide direct evidence that Cyclin K plays a critical role in the DNA damage response.

### Knockdown of Cyclin K impairs radioresistance in lung cancer *in vitro* and *in vivo*

**Figure 4:**
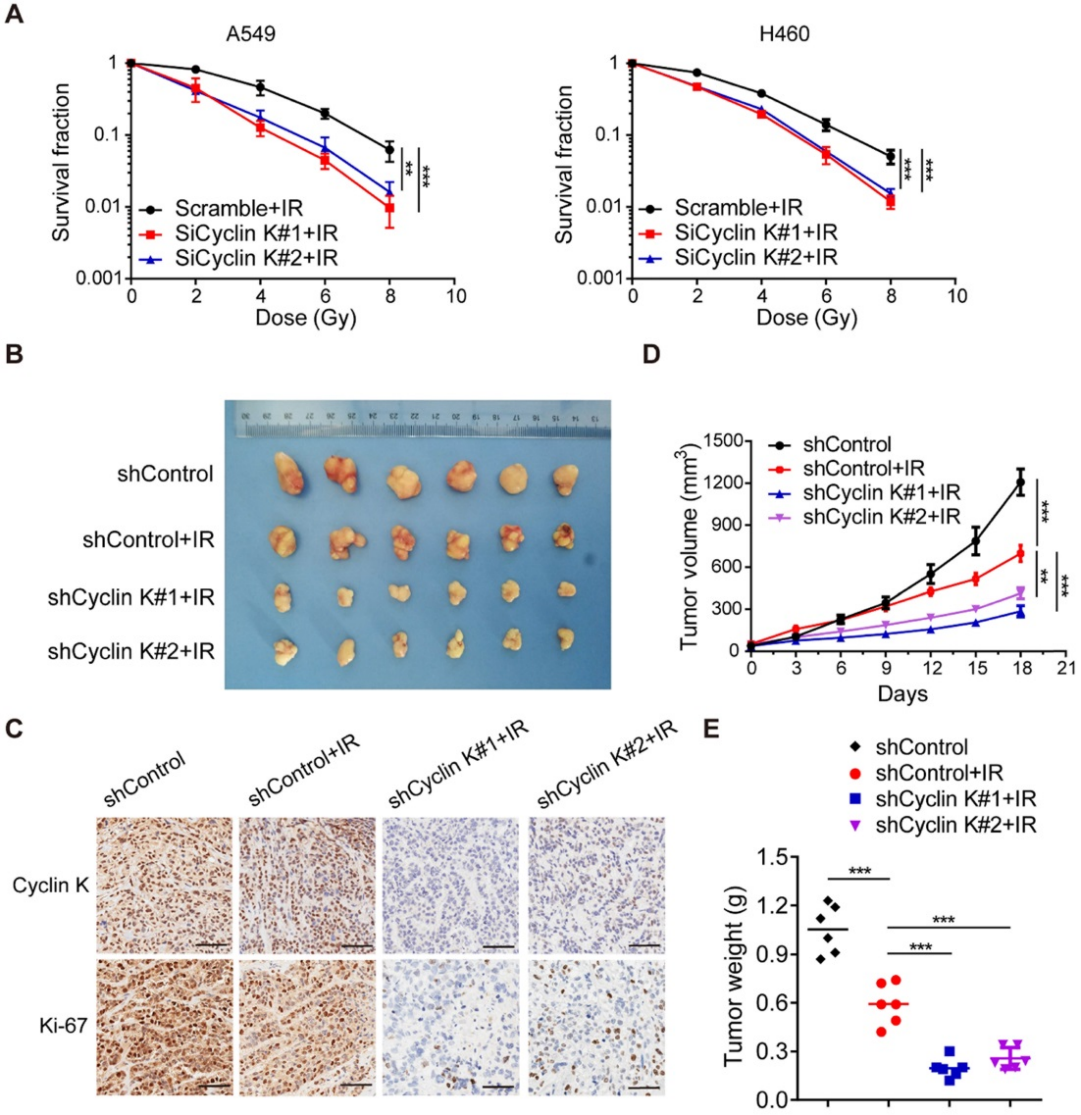
** Loss of Cyclin K significantly impairs radioresistance in lung cancer* in vitro* and *in vivo*. (A)** Cyclin K depletion led to radiosensitivity *in vitro*. Cells were transfected with indicated siRNAs and irradiated with doses as indicated. Colonies were counted after fourteen days. **P < 0.01. ***P < 0.001 (n = 3). **(B)** Cyclin K knockdown impaired radioresistance in lung cancer *in vivo*. Representative images of xenograft tumors in four groups are shown. Mice were subcutaneously injected in the right posterior limb with 5×10^6^ H460 cells as indicated and subjected to 10 Gy irradiation when tumors reached a calculated average volume of 100 mm^3^ (six mice per group). **(C)** Representative immunohistochemistry images showing Cyclin K and Ki-67 expression in xenograft tumors in the four groups. Scale bar, 50 µm. **(D)** Growth curves of the tumors in the four groups are presented (six mice/group). Tumor sizes were measured every three days using calipers. Data are shown as the mean tumor volume ± SEM. **(E)** Tumor weight in the four groups are shown (six mice/group).

To further explore whether Cyclin K directly controls cancer cell radiosensitivity, we performed clonogenic survival assays to ascertain cell survival after irradiation. As expected, loss of Cyclin K enhanced cellular sensitivity to radiation (Figure 4A). These findings indicate that Cyclin K depletion impairs radioresistance *in vitro*. Next, we examined the effect of Cyclin K knockdown on xenografts in nude mice treated with irradiation. As shown in Figure 4B-D, tumor size in the groups exposed to irradiation was significantly smaller than in control groups, suggesting that irradiation was effective. More strikingly, we found that both tumor size and weight in shCyclin K groups exposed to irradiation were smaller compared to shControl groups with irradiation (Figure 4B-E). Consistent with this notion, protein levels of Ki-67 in shCyclin K groups exposed to irradiation were significantly decreased (Figure 4C). These results indicate that Cyclin K silencing accelerates cellular DNA damage and contributes to enhanced radiosensitivity in lung cancer *in vitro* and *in vivo*.

### Cyclin K interacts with and enhances the stabilization of β-catenin protein to upregulate Cyclin D1 in lung cancer cells

**Figure 5:**
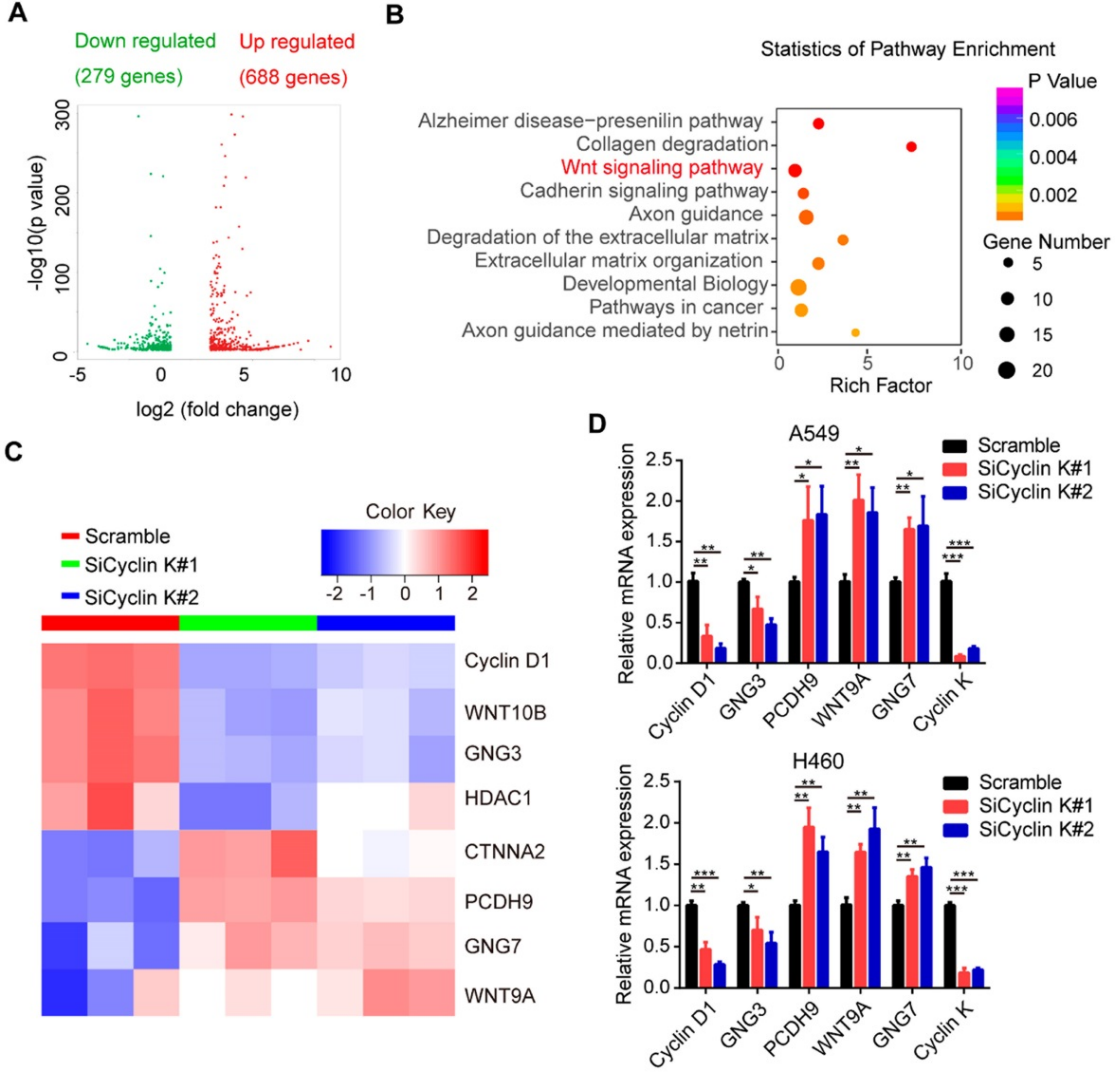
** Cyclin K is involved in the activation of the Wnt signaling pathway. (A)** Volcano plot showing upregulated (red) and downregulated (green) differentially expressed genes from RNA sequence analysis between scrambled and siCyclin K groups. (Fold change > 3 and *p*-value < 0.01).** (B)** Statistics of pathway analysis of differentially expressed mRNA transcripts identified by RNA-seq. **(C)** Heat map of representative mRNA changes in the Wnt signaling pathway by RNA-seq. **(D)** A549 and H460 cells were transfected with indicated siRNAs for 48 h and then collected. Levels of indicated mRNAs were determined using quantitative real-time PCR. *P < 0.05, **P < 0.01, ***P < 0.001 (n = 3).

To elucidate the underlying mechanisms by which Cyclin K functions in lung cancer, we performed RNA sequencing (RNA-Seq) in A549 cells transfected with scrambled or Cyclin K siRNA. As shown in Figure 5A, RNA-seq analysis identified 967 differentially expressed genes with at least two-fold change. Among them, 279 genes were significantly upregulated and 688 genes downregulated in Cyclin K depleted A549 cells. Next, we performed multiple pathway analysis and found that the Wnt signaling pathway, which is closely associated with tumor progression and radioresistance, was markedly enriched (Figure 5B), indicating that cyclin K may represent a critical regulator of Wnt signaling. To confirm this finding, we first performed quantitative real-time PCR to assess the expression of several representative genes involved in Wnt signaling (Figure 5C). As shown in Figure 5D, inhibition of Cyclin K with siRNA significantly reduced basal expression levels of Cyclin D1 and GNG3, while increasing expression levels of PCDH9, WNT9A and GNG7 in two different lung cancer cell lines.

**Figure 6:**
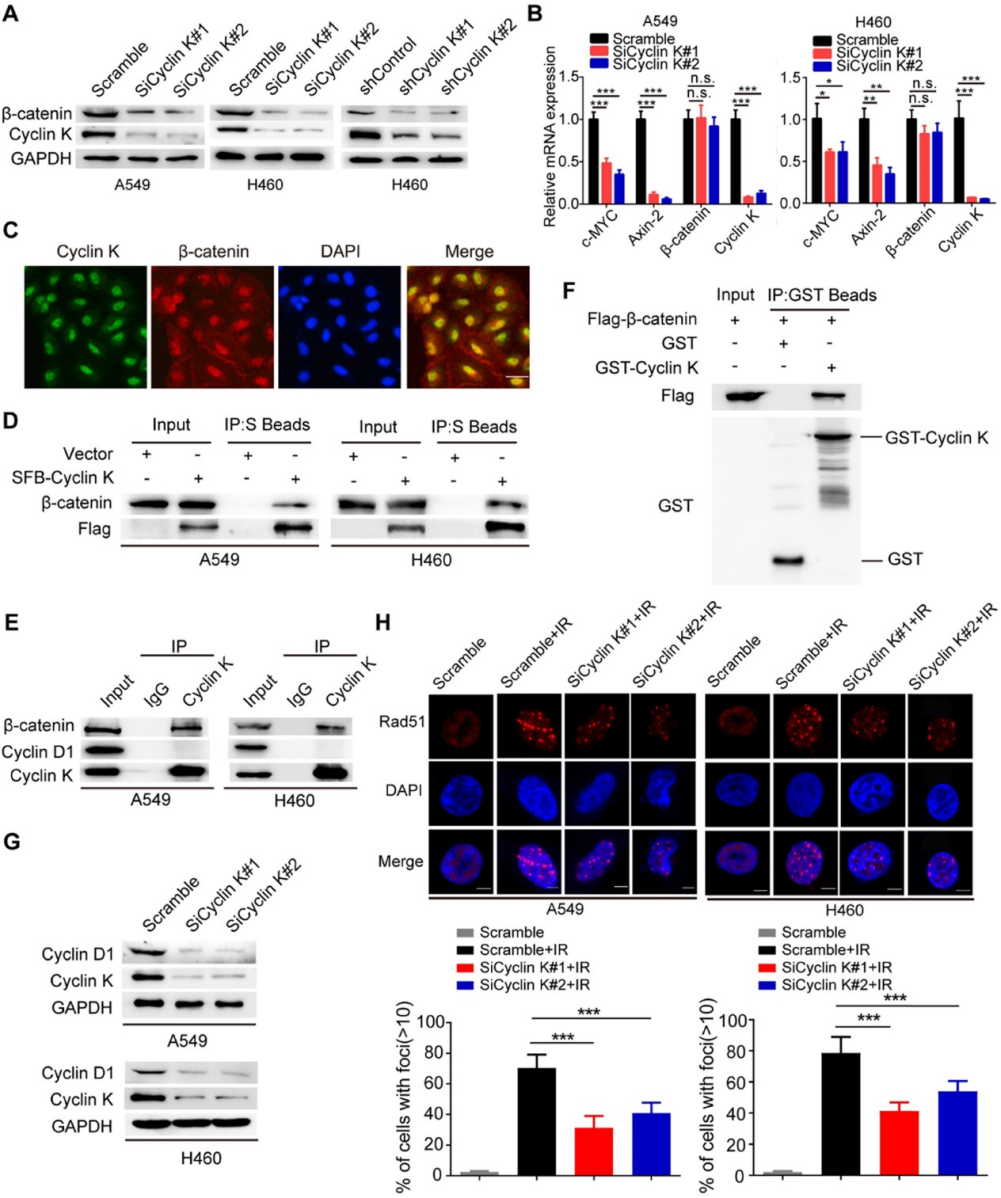
** Cyclin K interacts with and enhances the stabilization of β-catenin protein to upregulate Cyclin D1 in lung cancer cells. (A)** Cyclin K depletion using siRNAs or shRNAs led to decreased levels of β-catenin (n = 3). **(B)** A549 and H460 cells were transfected with indicated siRNAs for 48 h and then harvested. Levels of indicated mRNAs were detected using quantitative real-time PCR. *P < 0.05, **P < 0.01, ***P < 0.001 (n = 3). n. s. means no statistically significant difference (*P* > 0.05). **(C)** Subcellular localization of endogenous Cyclin K (green) and β-catenin (red) in paraformaldehyde-fixed A549 cells (n = 3). Scale bar, 50 µm.** (D)** Exogenous Cyclin K binds to endogenous β-catenin. A549 and H460 cells were transfected with the indicated construct for 24 h. Co-IP experiments were performed using S-protein beads and blotted with the indicated antibodies (n = 3). **(E)** Endogenous interaction between Cyclin K and β-catenin was detected in A549 and H460 cells (n = 3). IgG was used as a negative control. **(F)** Beads coated with the bacterially expressed GST-vector or GST-Cyclin K were incubated with cell lysates from HEK293T cells transfected with Flag-β-catenin overnight. Samples were immunoblotted with the indicated antibodies (n = 3).** (G)** Protein levels of Cyclin D1 were decreased in Cyclin K-depleted lung cancer cells (n = 3). **(H)** Upper panel: Cells transfected with indicated siRNAs were irradiated with 6 Gy and then collected after 4 h. Representative immunostaining images are shown. Lower panel: Quantification results of Rad51 foci are shown. Scale bar, 10 µm. ***P < 0.001 (n = 3).

β-catenin is a vital effector of the canonical Wnt signaling pathway and is overexpressed in lung cancer 29. To further investigate whether Cyclin K regulates Wnt signaling, we examined expression levels of β-catenin in Cyclin K knockdown cells. As expected, protein levels of β-catenin were decreased when Cyclin K was depleted with siRNA or shRNA (Figure 6A). Notably, Cyclin K depletion did not affect mRNA levels of β-catenin (Figure 6B). However, we found that mRNA levels of other classical target genes of β-catenin, such as C-MYC and Axin-2, were downregulated in Cyclin K deficient cells (Figure 6B). To further investigate how Cyclin K controls β-catenin activity, we first performed immunofluorescence assays and found that Cyclin K co-localized with β-catenin in the nuclei (Figure 6C), suggesting that these two proteins may bind to each other in cells. As expected, a clear interaction was observed between Cyclin K and β-catenin (Figure 6D). Moreover, Cyclin K and β-catenin were able to form a complex endogenously (Figure 6E). In addition, GST pull-down assay revealed that bacterially expressed GST-tagged Cyclin K bound to β-catenin (Figure 6F). These results indicate that Cyclin K binds to and controls the protein expression of β-catenin in lung cancer. It is well-known that Cyclin D1 is a representative target gene of β-catenin and involved in the regulation of cell proliferation and DNA damage response 30, 31. Our results clearly showed that Cyclin K knockdown led to decreased protein levels of Cyclin D1 (Figure 6G). Cyclin D1 has been reported to recruit Rad51, a protein that plays an essential role in the homologous recombination process at the DNA double-strand break site 30. Therefore, we performed immunofluorescence staining and found that IR-induced Rad51 foci formation was impaired when Cyclin K was depleted (Figure 6H). Collectively, these results indicate that Cyclin K interacts with and promotes the stabilization of β-catenin, thereby upregulating the expression of Cyclin D1 and facilitating DNA repair in lung cancer.

### Cyclin D1 is required for the biological functions of Cyclin K in lung cancer cells

**Figure 7:**
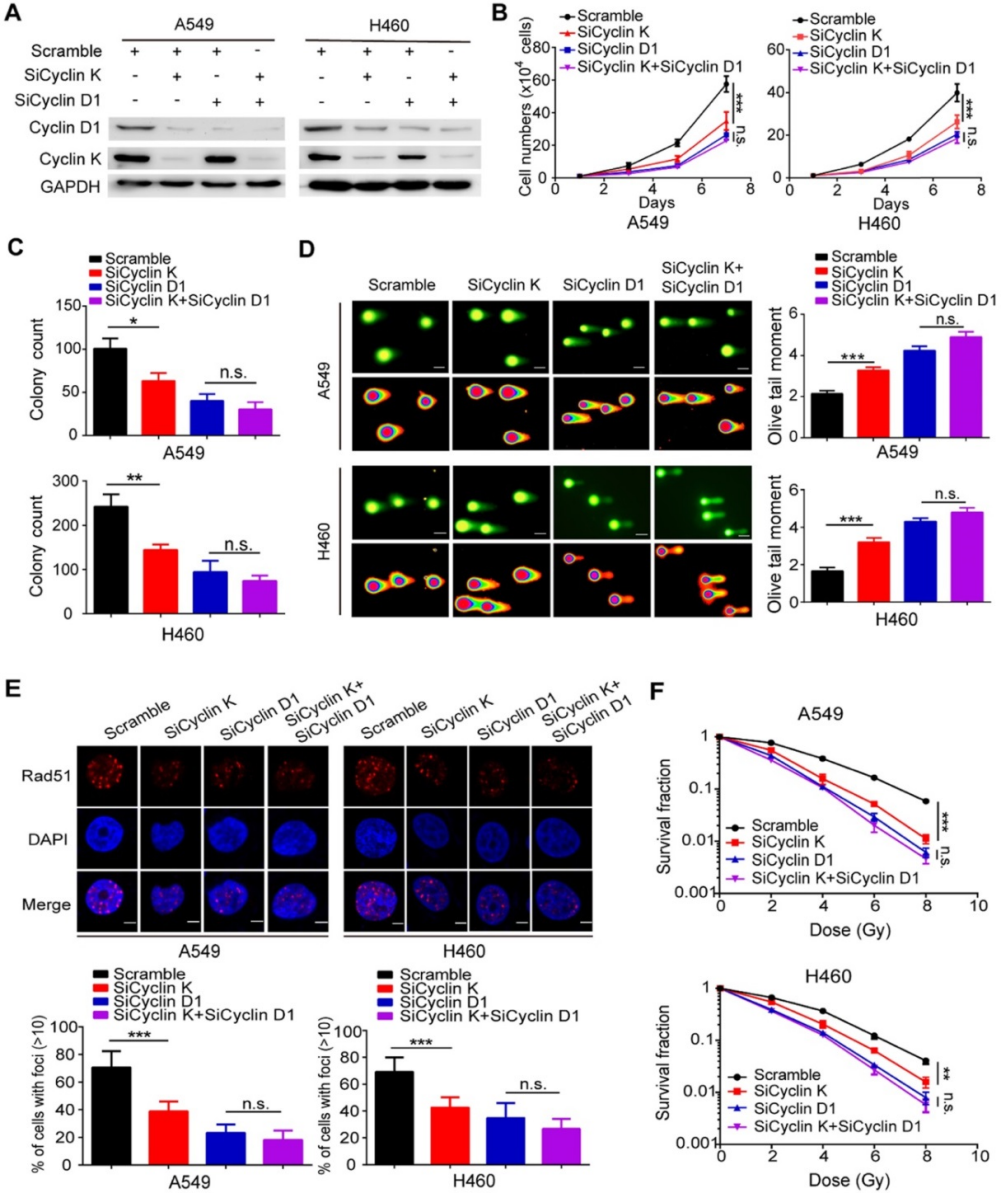
** Cyclin D1 mediates the biological effects of Cyclin K depletion in lung cancer cells. (A)** Cells were transfected with the indicated siRNAs for 48 h and then harvested and analyzed by Western blotting (n = 3). **(B)** Cells transfected with the indicated siRNAs were seeded at low density, and cell numbers were counted every other day. **P < 0.01 (n = 3). n. s. means no statistically significant difference (*P* > 0.05). **(C)** Cells were transfected with the indicated siRNAs and grown for two weeks. Quantification results of colony formation are shown. *P < 0.05, **P < 0.01 (n = 3). **(D)** Cells were transfected with the indicated siRNA and then exposed to IR (6 Gy). After 4 h, cells were harvested and seeded onto comet slides and then lysed for electrophoresis. ***P < 0.001(n = 3), Scale bar, 10 µm. **(E)** Upper panel: Cyclin K-depleted cells were irradiated with 6 Gy and harvested after 4 h. Representative immunostaining images are shown. Lower panel: Quantification results of Rad51 foci are also shown. ***P < 0.001(n = 3), Scale bar, 10 µm.** (F)** Cells were transfected with the indicated siRNAs and irradiated with doses as indicated. Colonies were counted after two weeks. **P < 0.01, ***P < 0.001(n = 3).

To determine whether Cyclin D1 is indeed required for the observed phenotypes induced by Cyclin K silencing in lung cancer cells, the following rescue experiments were conducted. A549 and H460 cells were transfected with siRNAs targeting Cyclin K, Cyclin D1 or both (Figure 7A). As shown in Figure 7B-C, loss of Cyclin K or Cyclin D1 markedly suppressed lung cancer cell growth and proliferation. However, Cyclin K knockdown had minimal effects on cell growth and proliferation in Cyclin D1-depleted cells (Figure 7B-C), indicating that Cyclin D1 is the major downstream effector of Cyclin K. Furthermore, we observed that Cyclin D1 depletion accelerated DNA damage, impaired Rad51 foci formation and enhanced radiosensitivity in lung cancer cells (Figure 7D-F), consistent with previous studies showing that Cyclin D1 is a critical regulator of DNA damage repair. Notably, our results demonstrated that further inhibition of Cyclin K with siRNA in lung cancer cells led to insignificant effects on these phenotypes when Cyclin D1 was simultaneously knocked down (Figure 7D-F). These data demonstrate that Cyclin K promotes tumorigenesis and radioresistance in a Cyclin D1-dependent manner in lung cancer.

**Figure 8:**
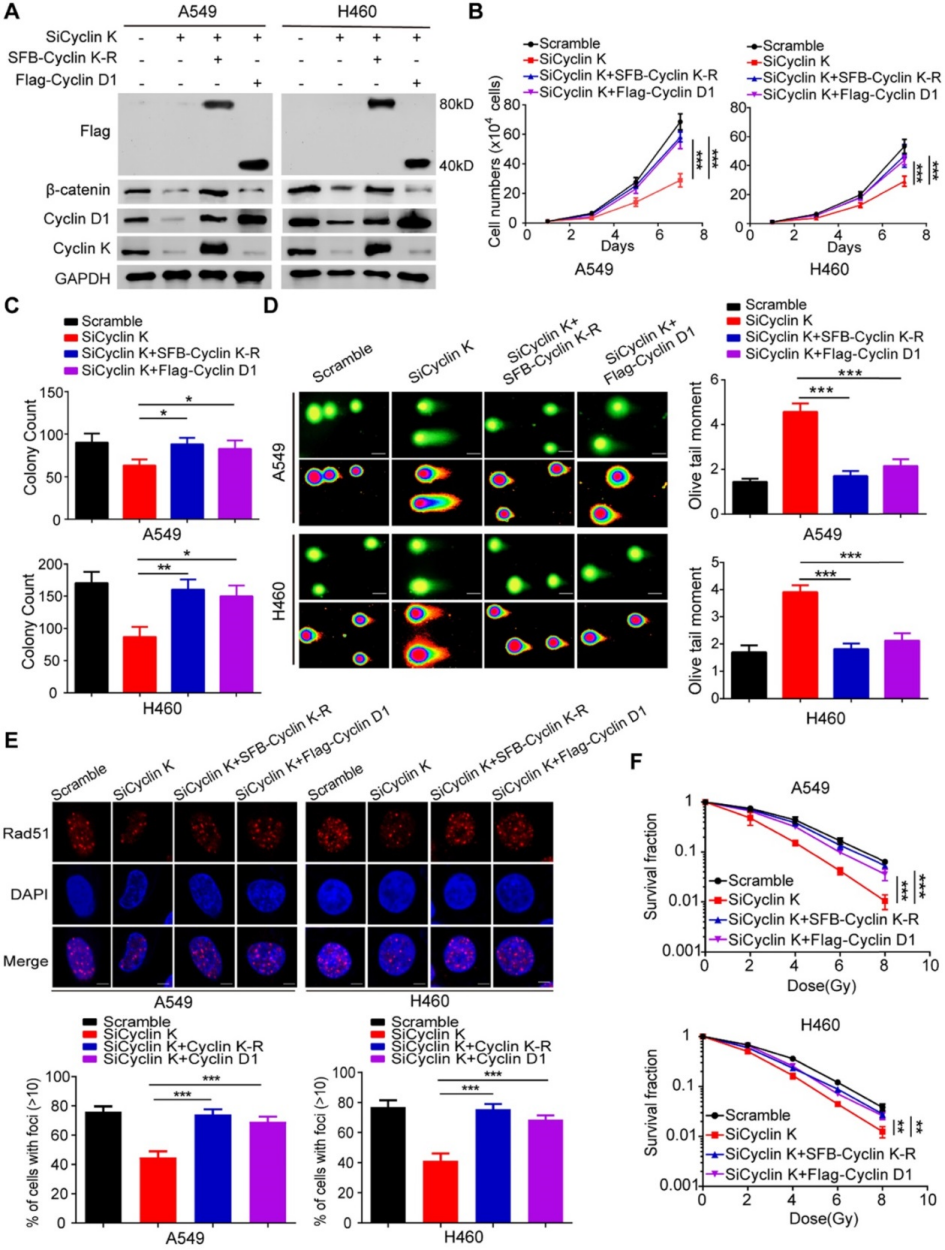
** Cyclin D1 overexpression partially rescued the biological effects of Cyclin K depletion in lung cancer cells. (A)** Cells transfected with indicated siRNAs and plasmids were harvested and analyzed by Western blotting (n = 3). **(B)** Cells transfected with the indicated siRNAs and plasmids were seeded at low density. Cell numbers were counted every other day. ***P < 0.001(n = 3). **(C)** Cells transfected with the indicated siRNAs and plasmids were cultured for two weeks. Quantification results of colony formation are shown. *P < 0.05, **P < 0.01 (n = 3). **(D)** Cells transfected with indicated siRNAs and plasmids were exposed to IR. After 4 h, cells were harvested, and comet assay was conducted. ***P < 0.001(n = 3), Scale bar, 10 µm. **(E)** Upper panel: Representative immunostaining images of Rad51 foci are shown. Lower panel: Quantification results of Rad51 foci are also presented. ***P < 0.001 (n = 3), Scale bar, 10 µm.** (F)** Cells transfected with indicated siRNAs and plasmids were irradiated with indicated doses. Colonies were quantified after two weeks. **P < 0.01, ***P < 0.001 (n = 3).

To exclude any off-target effects of Cyclin K siRNA and to further confirm the roles of Cyclin K in lung cancer, we generated a Cyclin K siRNA resistant plasmid (SFB-Cyclin K-R) (Figure 8A). As shown in Figure 8B-C, cell growth and proliferation defects caused by Cyclin K knockdown were completely rescued by re-expressing Cyclin K. Similarly, the reconstitution of Cyclin K completely restored the IR-induced DNA damage response in Cyclin K-depleted cells (Figure 8D-F). These data demonstrate that Cyclin K is indeed implicated in lung cancer progression and radioresistance. Moreover, to further investigate whether Cyclin D1 is required for the biological functions of Cyclin K in lung cancer, we transfected exogenously expressed Cyclin D1 into Cyclin K-depleted cells and found that the restoration of Cyclin D1 partially reversed the effects of Cyclin K silencing on cell growth suppression and radiosensitivity (Figure 8B-F). Taken together, our work supports the notion that Cyclin K exerts its biological functions primarily through regulating Cyclin D1 in lung cancer cells.

## Discussion

In this study, we found that Cyclin K is overexpressed in lung cancer patients, and high levels of Cyclin K predict poor prognosis. We also provided strong evidence that Cyclin K knockdown impairs tumorigenesis and radioresistance in lung cancer both *in vitro* and *in vivo* through attenuating Wnt/β-catenin signaling. Furthermore, we revealed that the β-catenin target gene Cyclin D1 is an essential downstream effector that mediates the biological effects of Cyclin K, implying that the Cyclin K/β-catenin/Cyclin D1 pathway represents a potential therapeutic target in lung cancer.

Unlike classical cell cycle-related cyclins, Cyclin K is regarded as a member of the “transcription” cyclin family that participates in RNA polymerase II transcription 7-9. Accumulating evidence suggests that Cyclin K functions primarily through forming a complex with other proteins. For instance, Cyclin K interacts with CDK9 to activate transcription and is involved in the replication stress response 8, 9. Cyclin K has also been reported to associate with CDK12/CDK13 and controls the expression of several critical regulators of genome integrity, including BRCA1, ATR and FANCI proteins 10. Recently, the H3K4 methyltransferase SETD1A has been proposed to regulate the expression of genes involved in DNA damage response by interacting with cyclin K, and this interaction is required for leukemia cell growth 14. In our study, we identified β-catenin as a novel Cyclin K binding partner in lung cancer. Cyclin K forms a complex with β-catenin and enhances its protein stabilization, inducing the expression of downstream target genes of β-catenin, especially Cyclin D1. Furthermore, we also revealed that Cyclin K controls protein levels but not mRNA levels of β-catenin, suggesting that Cyclin K may positively regulate β-catenin at the post-transcriptional level. Previous studies have demonstrated that β-catenin is a labile protein that can be ubiquitylated and degraded by the proteasome 15-17. Therefore, we propose that Cyclin K interacts with β-catenin and contributes to its protein stabilization by protecting β-catenin from ubiquitin-mediated degradation, which requires further investigation in the future. A growing body of evidence indicates that β-catenin is the key effector of the canonical Wnt pathway and mediates many cellular processes in response to Wnt signaling 17. Using RNA-Seq screening and pathway enrichment analysis, we found that Wnt/β-catenin signaling is the most significantly altered cancer-related pathway when Cyclin K was depleted. Furthermore, bioinformatics analysis with TCGA database demonstrated that Cyclin K expression is positively correlated with both β-catenin and Cyclin D1 levels in lung cancer tissues (Figure S2). Therefore, these results strongly suggest that Cyclin K is a newly identified regulator of Wnt/β-catenin signaling that positively modulates the nuclear β-catenin, providing new insights into the mechanisms by which Cyclin K functions in lung cancer.

In addition to regulating gene transcription, Cyclin K has also been implicated in the control of cell proliferation and apoptosis, indicating that Cyclin K may play indispensable roles in tumor development and progression. For example, Cyclin K regulates Aurora B expression to affect apoptosis and proliferation by inducing mitotic catastrophe in prostate cancer 32. In addition, a recent study showed that Cyclin K promotes mammalian cell proliferation by controlling prereplicative complex assembly 33. However, the functional role of Cyclin K in cancer is still poorly understood. In our study, we uncovered for the first time that Cyclin K is frequently overproduced in lung cancer and is associated with overall survival in lung cancer patients. Additionally, we present evidence that Cyclin K silencing significantly reduced cell growth and proliferation both *in vitro* and *in vivo*, and these effects are primarily mediated by Cyclin D1. These results indicate that Cyclin K exerts oncogenic effects in lung cancer. More importantly, we also revealed a novel role for Cyclin K in the regulation of lung cancer radioresistance. Cyclin K depletion abolished the G2/M checkpoint, aggravated cellular DNA damage and impaired DNA repair capacity, ultimately leading to enhanced radiosensitivity by downregulating the expression of the β-catenin target gene Cyclin D1. Therefore, our data indicate that Cyclin K plays pivotal roles in promoting tumorigenesis and radioresistance in lung cancer.

In conclusion, our study identifies a novel interaction between Cyclin K and β-catenin that comprises a critical effector of Wnt signaling. Functionally, we demonstrate that Cyclin K is implicated in tumorigenesis and radioresistance primarily via positively regulating the Wnt/β-catenin/Cyclin D1 signaling pathway. Given that Cyclin K is overexpressed in lung cancer tissues and is related to radioresistance, it may represent a promising therapeutic biomarker for cancer radiotherapy, especially in lung cancer.

## Supplementary Material

Supplementary figures and tables.

## Abbreviations

NSCLC: Non-small cell lung cancer
CDKs: Cyclin-dependent kinases
CKI: CDK inhibitors
RNAP II: RNA polymerase II
NHEJ: non-homologous end joining
FBS: fetal bovine serum
DMEM: Dulbecco's modified Eagle medium
pH3: phospho-Ser10-histone H3
DDR: DNA damage response
IP: immunoprecipitation
IHC: immunohistochemical
RNA-Seq: RNA sequencing
OS: overall survival
CFSE: carboxy fluorescein succinimidyl ester
