# Supplementary material for: Cyclin K interacts with β-catenin to induce Cyclin D1 expression and facilitates tumorigenesis and radioresistance in lung cancer
Source: Theranostics. 2020 Sep 11;10(24):11144–58. doi: 10.7150/thno.42578 (PMC7532664; doi:10.7150/thno.42578)
Supplement: Supplementary file 1 — Supplementary figures and tables. [file thnov10p11144s1.pdf]

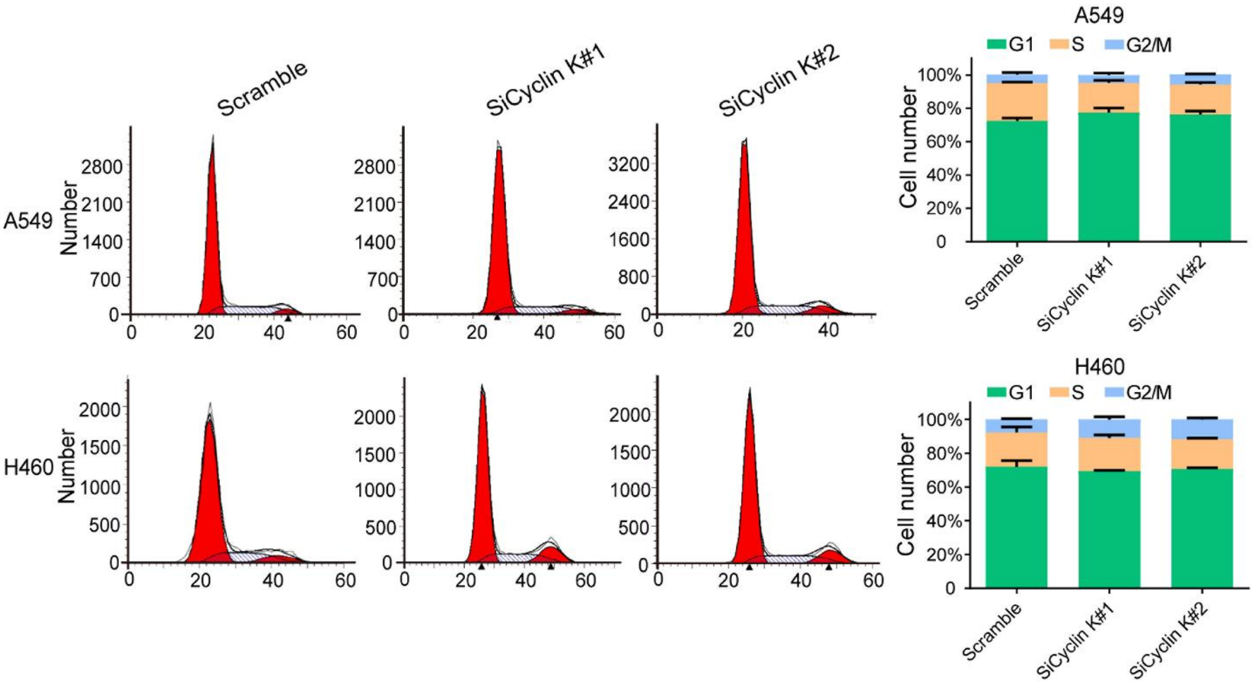

Figure S1. A549 and H460 cells transfected with indicated siRNAs for 48 h were collected and analyzed by flow cytometry (n = 3).

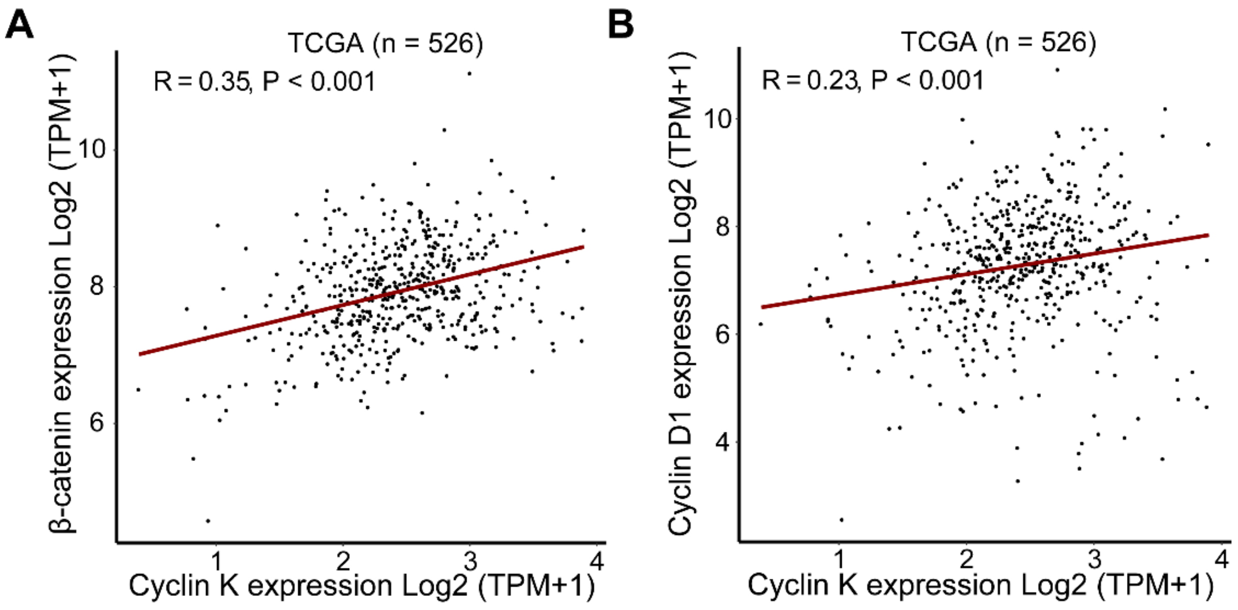

Figure S2. Scatterplots of Cyclin K vs β-catenin and Cyclin D1 mRNA expression in lung cancer samples available from TCGA database (n = 526). The Pearson correlation coefficient (R) and p value are shown.

Genes	Sequences (5'--3')	Size（bp）
Cyclin K	F [a]: AAATGACAGTCTCTGCACCACCTT<br>R: ACAAGAATGGACCTCCAAACCCAT	77
Cyclin D1	F: CCTCTGTGCCACAGATG<br>R: GGGTCACACTTGATCACTC	144
GNG3	F: GATAAAGGTGTCCAAGGCAGC<br>R: AACTTCTTCTCCCGGAAGGG	95
PCDH9	F: CAGGAACATAGGGGATAGTAGCC<br>R: CACCAGAACGGTGACGAAGA	94
WNT9A	F: GGACTTCCACAACAACCTCG<br>R: GTGCCGTCTCATACTTGTGCT	146
GNG7	F: ACTAACAACATAGCCCAGGCC<br>R: CTTAAAGGGGTTCTCCGAGG	149
β-catenin	F: CAACTAAACAGGAAGGGATGGAAGG<br>R: CAGATGACGAAGAGCACAGATGG	213
c-MYC	F: CAGCGACTCTGAGGAGGAAC<br>R: TGTGAGGAGGTTTGCTGTGG	110
Axin-2	F: TGACGGACAGCAGTGTAGATG<br>R: GGTTCTCGGGAAATGAGGTAG	103
GAPDH	F: AGAAGGCTGGGGCTCATTTG<br>R: AGGGGCCATCCACAGTCTTC	137

[a] F, forward primer; R, reverse primer.

Table S1. Sequences of primers used for Real-time quantitative PCR.

Features	Cyclin K expression		P value
	high	low	
**Mean age (years)**			
61.915	65	17	
**Age(years)**			
≤60	34	11	0.421
＞60	31	6	
**Sex**			
Male	37	7	0.284
Female	28	10	
**Tumor size**			
T1(≤3cm)	21	9	0.290
T2(＞3cm，≤7cm)	38	7	
T3（＞7cm）	6	1	
**Stage**			
Ⅰ	19	6	**0.024**
Ⅱ	14	9	
Ⅲ	31	2	
Ⅳ	1	0	
**T**			
1	12	5	0.479
2	37	10	
3	10	2	
4	6	0	
**N**			
0	26	6	0.954
1	12	3	
2	13	2	
3	3	1	
**M**			
0	64	17	0.607
1	1	0	

Table S2. Association between the clinicopathologic parameters and expression of Cyclin K in human lung cancer tissues.
